# The Delta Study – Prevalence and characteristics of mood disorders in 924 individuals with low mood: Results of the of the World Health Organization Composite International Diagnostic Interview (CIDI)

**DOI:** 10.1002/brb3.2167

**Published:** 2021-05-07

**Authors:** Nayra A. Martin‐Key, Tony Olmert, Giles Barton‐Owen, Sung Yeon Sarah Han, Jason D. Cooper, Pawel Eljasz, Lynn P. Farrag, Lauren V. Friend, Emily Bell, Dan Cowell, Jakub Tomasik, Sabine Bahn

**Affiliations:** ^1^ Department of Chemical Engineering and Biotechnology University of Cambridge Cambridge UK; ^2^ Psyomics Ltd Cambridge UK

**Keywords:** bipolar disorder, diagnostic errors, major depressive disorder, mood disorders, prevalence

## Abstract

**Objectives:**

The Delta Study was undertaken to improve the diagnosis of mood disorders in individuals presenting with low mood. The current study aimed to estimate the prevalence and explore the characteristics of mood disorders in participants of the Delta Study, and discuss their implications for clinical practice.

**Methods:**

Individuals with low mood (Patients Health Questionnaire‐9 score ≥5) and either no previous mood disorder diagnosis (baseline low mood group, *n* = 429), a recent (≤5 years) clinical diagnosis of MDD (baseline MDD group, *n* = 441) or a previous clinical diagnosis of BD (established BD group, *n* = 54), were recruited online. Self‐reported demographic and clinical data were collected through an extensive online mental health questionnaire and mood disorder diagnoses were determined with the World Health Organization Composite International Diagnostic Interview (CIDI).

**Results:**

The prevalence of BD and MDD in the baseline low mood group was 24% and 36%, respectively. The prevalence of BD among individuals with a recent diagnosis of MDD was 31%. Participants with BD in both baseline low mood and baseline MDD groups were characterized by a younger age at onset of the first low mood episode, more severe depressive symptoms and lower wellbeing, relative to the MDD or low mood groups. Approximately half the individuals with BD diagnosed as MDD (49%) had experienced (hypo)manic symptoms prior to being diagnosed with MDD.

**Conclusions:**

The current results confirm high under‐ and misdiagnosis rates of mood disorders in individuals presenting with low mood, potentially leading to worsening of symptoms and decreased well‐being, and indicate the need for improved mental health triage in primary care.

## Graphical abstract

The Delta Study was undertaken to improve the diagnosis of mood disorders in individuals presenting with low mood. The current study aimed to estimate the prevalence and explore the characteristics of mood disorders in participants of the Delta Study, and discuss their implications for clinical practice. This study confirms high under‐ and misdiagnosis rates of mood disorders in individuals presenting with low mood, potentially leading to worsening of symptoms and decreased wellbeing, and indicate the need for improved mental health triage in primary care.

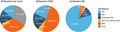

## 1 INTRODUCTION

Major depressive disorder (MDD) and bipolar disorder (BD) are complex and debilitating conditions affecting 16.2% and 2.4% of the population worldwide, respectively (Merikangas et al., 2011; Kupfer et al., 2012), and are among the leading contributors to the global burden of diseases (GBD, 2020). Core symptoms of MDD include a pervasive and persistent disturbance of mood and loss of interest/pleasure in most daily activities (Otte et al., 2016). Individuals can also experience impaired concentration and indecisiveness, as well as fatigue or low energy, disturbances to sleep and appetite, headaches, muscle tension, and general symptoms of pain (American Psychiatric Association, 2013). BD, on the other hand, is typically characterized by intermittent depressive and manic (BDI) or hypomanic (BDII) episodes. While depressive episodes in BD may be indistinguishable from those in MDD, (hypo)manic episodes can include elevated levels of energy, euphoric mood, irritability, and hypersexuality, as well as a reduced need for sleep (Einat, 2007). Diagnosing MDD and BD requires a comprehensive collection of symptom‐ and patient‐level data, as well as information on family history, course of illness, and prior treatment response. The World Health Organization World Mental Health Composite International Diagnostic Interview (CIDI (Kessler & Üstün, 2004)) can be a useful tool for the assessment and differential diagnosis of MDD and BD. It also captures subthreshold forms of elevated mood, including subthreshold BD and MDD with subthreshold BD, with individuals presenting with fewer and/or a shorter duration of (hypo)manic symptoms.

Both MDD and BD typically start early in life and are associated with severe functional impairments, high morbidity and mortality, including premature death due to suicide (Bourne et al., 2013; Passos et al., 2016; Ösby et al., 2016). The economic burden of MDD and BD is also substantial; in England, direct and indirect annual costs are estimated at £7.46 billion (US $9.85 billion) for MDD and £5.25 billion (US $6.93 billion) for BD (McCrone et al., 2008). In spite of their prevalence and negative prognosis, the recognition and diagnosis of these conditions presents a significant challenge, particularly in the primary care setting. For instance, research has shown that general practitioners (GPs) initially misdiagnose 50% of MDD patients (Mitchell et al., 2009). Short consultation times coupled with the difficulties associated with diagnosing MDD, where any two individuals may have no symptoms in common (Olbert et al., 2014; Fried et al., 2014), means that many are not receiving the support they need. This is a particular issue, given that the vast majority of patients with MDD receive treatment and care solely in the primary care setting.

In the case of BD, 60% of individuals are initially misdiagnosed with MDD (Hirschfeld et al., 2003), with many having to wait 8 to 10 years before receiving a correct diagnosis (Bauer et al., 2018; Patel et al., 2015). This is due, in part, to the fact that the majority of individuals with BD seek help when they are experiencing depressive symptoms as opposed to when they are in a (hypo)manic state. Furthermore, in most instances, depressive episodes precede a first (hypo)manic episode, and awareness of one's (hypo)manic symptoms is relatively low (Regeer et al., 2015). This poses a significant problem for the diagnosis, treatment, and management of BD, with these individuals likely to be treated with antidepressant monotherapy, which is frequently ineffective in treating bipolar depression. Critically, the use of antidepressants without a concurrent mood stabilizer can trigger and exacerbate a hypo(manic) episode, resulting in prolonged suffering and, in some cases, suicide (Bowden, 2005).

Taken together, the careful evaluation and management of *all* patients presenting with depressive symptoms is warranted. Even in the absence of a (hypo)manic episode, individuals diagnosed with MDD should be closely monitored and managed. This is because symptoms of MDD are frequently the initial presentation of BD, with factors including greater depression severity, recurrent MDD, and psychotic symptoms associated with a later BD diagnosis (Holma et al., 2008). Other, patient‐level risk factors that are indicative of the disorder comprise an earlier age of onset of depressive symptoms, being white, living alone, not being married, and being unemployed (Hirschfeld et al., 2005). While the collection of extensive symptom‐ and patient‐level data may prove difficult in the primary care setting, where time is a luxury, digital technologies may offer an innovative, time‐ and cost‐effective alternative to conventional, interview‐based methods.

A comprehensive and careful appraisal of the characteristics of individuals presenting with depressive symptoms is likely to improve biological disease understanding, facilitate patient stratification, and allow for personalized treatment plans and strategies. To this end, we carried out the Delta Study (Olmert et al., 2020), which aimed to develop and validate diagnostic algorithms, based on an online mental health questionnaire and blood biomarker data, that would reduce the misdiagnosis of BD as MDD as well as achieve a more accurate and timely diagnosis of MDD in those presenting with depressive symptoms. We adapted voluntary response sampling and online advertising to meet study recruitment targets estimated from published reports (Benazzi, 1997; Hantouche et al., 1998; Zuithoff et al., 2010; Sung et al., 2013). The present study examined the resulting prevalence and characteristics of mood disorders in the Delta Study population, determined using the CIDI, and their implications for clinical practice.

## 2 PARTICIPANTS AND METHODS

### 2.1 Study design and participants

**FIGURE 1:**
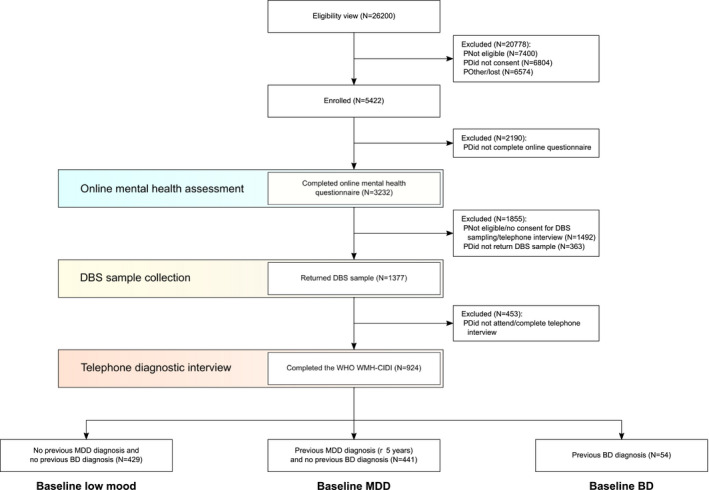
Delta Study flow diagram. Figure shows the number of individuals who completed each step of the study and reasons for attrition. BD, bipolar disorder; DBS, dried blood spot; MDD, major depressive disorder; WHO WMH‐CIDI, World Health Organization World Mental Health Composite International Diagnostic Interview

Data shown in the present work were collected as part of the Delta Study (International Registered Report Identifier RR1‐10.2196/18453), an investigator‐led study conducted by the Cambridge Centre for Neuropsychiatric Research (CCNR) at the University of Cambridge, which aimed to improve mood disorder diagnoses in participants presenting with depressive symptoms (Olmert et al., 2020). The primary objective of the Delta Study was to identify BD patients among patients diagnosed as having MDD. The secondary objective of the Delta Study was to identify patients with MDD among undiagnosed low mood individuals. To this end, three patient groups were recruited. The first group comprised patients with current depressive symptoms who had recently (≤5 years) (Ghaemi et al., 1999; Morselli et al., 2003) been diagnosed with MDD, the second group comprised participants with current depressive symptoms and no lifetime mood disorder diagnosis, and the third group comprised patients with current depressive symptoms and a previous lifetime BD diagnosis (Figure 1). The study was approved by the University of Cambridge Human Biology Research Ethics Committee (approval number HBREC 2017.11) and was conducted in compliance with the Declaration of Helsinki (World Medical Association, 2013), Good Clinical Practice, and ISO 14155:2011. A detailed research protocol for the Delta Study has been published previously (Olmert et al., 2020). Participants were recruited online through email, via the CCNR website, and Facebook. Inclusion criteria for the study were as follows: age 18 to 45 years, residency in the United Kingdom, at least mild depressive symptoms in the past two weeks (Patient Health Questionnaire (PHQ)‐9 (Kroenke et al., 2001) total score ≥5), not pregnant or breastfeeding, and not currently suicidal. All participants read the participant information sheet and digitally provided informed consent for participation in the study. Recruitment started on April 27, 2018 and was completed on September 28, 2018. The current report was prepared in compliance with the Strengthening the Reporting of Observational Studies in Epidemiology (STROBE) (Elm et al., 2007) guidelines.

### 2.2 Online mental health questionnaire

Upon enrolment, participants were asked to complete a purpose‐built online mental health questionnaire available through the Delta Study website. The online mental health questionnaire was developed in collaboration with experienced psychiatrists and a service user advisory group, and was based on existing structured diagnostic interviews as well as a range of mental health screening questionnaires. It consisted of 635 distinct questions divided into six modules: i) demographic information; ii) manic and hypomanic symptoms; iii) depressive symptoms; iv) personality traits; v) psychiatric history and vi) other psychiatric conditions. Participant wellbeing in the past two weeks was assessed using the Warwick‐Edinburgh Mental Wellbeing Scale (WEMWBS) (Tennant et al., 2007). The online mental health questionnaire was adaptive to answers given by participants, so that only relevant questions were asked, and the maximum possible number of questions asked to an individual was 382 (284 on average). Data collected from the online mental health questionnaire were used to identify participants qualifying for the study's primary and secondary objectives following a pre‐defined study protocol. Among the identified participants, those eligible for the diagnostic interview had to: i) consent to providing a blood sample (self‐collected dried blood spots) and completing a telephone diagnostic interview; ii) be free from blood‐borne illnesses; and iii) have no previous diagnosis of schizophrenia.

### 2.3 Diagnostic interview

Participants who successfully completed the online mental health questionnaire and returned a dried blood spot (DBS) sample were invited to complete the CIDI, version 3.0 (Kessler & Üstün, 2004) via telephone. The CIDI is a modular diagnostic tool which is widely used in epidemiological studies on mental health (Kessler et al., 2005). It shows good concordance with structured diagnostic interviews conducted by clinicians, such as the Structured Clinical Interview for DSM disorders (SCID; area under the receiver operating characteristic curve (AUC) = 0.75–0.87 for MDD and 0.93–0.97 for BDI and II) (Haro et al., 2006; Kessler et al., 2006), and high interrater and test‐retest agreement (86%–100% for MDD and 87%–99% for BDI and II) (Wittchen, 1994). All interviewers conducting the CIDI received in‐person training from an external CIDI‐certified instructor, as well as internal training and mentoring. Only modules of the CIDI required for lifetime mood disorder diagnosis, that is the screening, depression, and mania sections, were implemented, resulting in six possible outcomes: BDI, BDII, subthreshold BD, MDD, MDD with subthreshold BD, and none, referred to as ‘low mood’ hereafter. We adopted voluntary response sampling, whereby the CIDI interviews continued until pre‐specified study recruitment targets were met.

### 2.4 Statistical analysis

Power calculations showed that a minimum of 300 participants with a recent diagnosis of MDD by a medical professional and 300 symptomatic participants with no baseline diagnosis of mood disorder were required. Additionally, we aimed to recruit 40 participants with a previous diagnosis of BD made by a medical professional to provide a validation group. Data processing and analysis were conducted in R version 4.0.2 (R Core Team, 2020). Group differences were tested for using the Kruskal–Wallis test for continuous variables and the chi‐squared test for categorical variables (R package ‘tableone’ (Yoshida et al., 2020)). Post hoc tests included pairwise group comparisons using Dunn's test (R package ‘FSA’ (Ogle et al., 2020)) for continuous variables and pairwise chi‐squared tests (R package ‘rcompanion’ (Mangiafico, 2020)) for categorical variables, with Bonferroni correction for multiple comparisons. Figures were prepared in R and Inkscape version 1.0.

## 3 RESULTS

**FIGURE 2:**
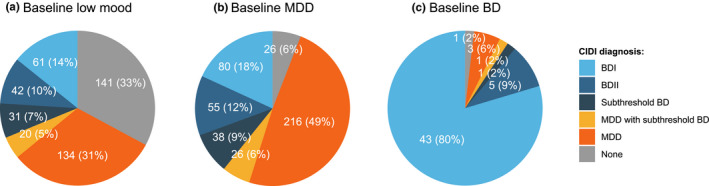
World Health Organization Composite International Diagnostic Interview (CIDI) outcomes across the Delta Study populations. The pie charts show the distribution of CIDI diagnoses in participants with (a) baseline depressive symptoms and no previous mood disorder diagnosis (*N* = 429), (b) baseline MDD diagnosis and no previous BD diagnosis (*N* = 441), and (c) baseline BD diagnosis (*N* = 54). BD, bipolar disorder; MDD, major depressive disorder

The study flow diagram is shown in Figure 1. To achieve study recruitment targets, 5,422 symptomatic individuals were enrolled, of which 3,232 completed the online mental health questionnaire and 924 completed the CIDI diagnostic interview. The average time interval between starting the online mental health questionnaire and completing the CIDI interview was 14 days. The dataset comprised of three groups: low mood individuals with no previous mood disorder diagnosis (*N* = 429); participants with a recent (≤5 years) diagnosis of MDD and no previous diagnosis of BD (*N* = 441); and participants with a previously established diagnosis of BD (*N* = 54; Figure 1). Of the 429 symptomatic participants with no previous diagnosis of mood disorder, the two largest groups comprised 154 participants (36%) with newly diagnosed MDD, including 20 individuals (5%) with concurrent subthreshold BD, and 141 (33%) low mood individuals (i.e., no mood disorder diagnosis by CIDI) (Figure 2a). The remaining diagnoses in the baseline low mood group included 103 (24%) diagnoses of BD, encompassing 61 participants (14%) with BDI and 42 participants (10%) with BDII, and 31 participants (7%) with subthreshold BD. Regarding the 441 participants with a recent diagnosis of MDD, the CIDI confirmed MDD in the majority of participants (*N* = 242; 55%), of which 26 (6%) had concurrent subthreshold BD (Figure 2b). Furthermore, 135 participants from the baseline MDD group (31%) were diagnosed with BD by the CIDI, including 80 participants (18%) with BDI and 55 participants (12%) with BDII. The remaining diagnoses in the baseline MDD group included 38 participants (9%) with subthreshold BD and 26 (6%) low mood individuals. In the validation group comprising participants with a previous diagnosis of BD, the CIDI showed 89% sensitivity in detecting BD (48/54; Figure 2c).

Table 1 and Appendix S1 show the characteristics of each of the diagnostic groups. Of those with no previous mood disorder diagnosis, individuals with BDI had a significantly different distribution of employment compared to the low mood group, with a higher proportion of professionals, and a lower proportion of students. Individuals with BDI were also more likely to have experienced childhood trauma relative to the low mood group. Participants with BDI scored lower in self‐rated quality of physical health relative to those with MDD and low mood. Additionally, individuals with BD and MDD scored lower in self‐rated quality of mental health relative to those with low mood. In comparison to the MDD and low mood groups, individuals with BDII were younger when they experienced their first depressive episode, and individuals with BDI and MDD reported having experienced a higher number of low mood episodes relative to the low mood group. As expected, individuals with BDI reported having experienced a higher number of episodes characterized by elevated mood in comparison to the MDD and low mood groups, and both the BDII and subthreshold BD groups experienced more elevated mood states relative to the low mood group. Finally, in comparison to low mood individuals, BD and MDD groups experienced more severe depressive episodes, as measured by the PHQ‐9, and reported poorer well‐being, as captured by the WEMWBS. No other significant group differences were observed in participants with no previous mood disorder diagnosis.

**TABLE 1:** Significant differences between the CIDI diagnostic groups in the Delta Study

Baseline diagnosis	Low mood						MDD					
CIDI diagnosis	BDI	BDII	MDD	None	*p*	Post hoc	BDI	BDII	MDD	None	*p*	Post hoc
*N*	61	42	134	141	NA	NA	80	55	216	26	NA	NA
Age, mean (*SD*), years	26.5 (6.3)	23.6 (4.4)	26.2 (6.2)	25.9 (6.6)	.143	NA	27.9 (7.5)	26.9 (6.7)	27.7 (6.8)	29.4 (7.2)	.671	NA
Sex, *N* (%)												
Male	27 (44)	12 (29)	40 (30)	50 (35)	.072	NA	33 (41)	20 (36)	56 (26)	9 (35)	.153	NA
Female	34 (56)	30 (71)	94 (70)	91 (65)			47 (59)	35 (64)	160 (74)	17 (65)		
Smoking^a^, *N* (%)												
No	21 (34)	16 (38)	74 (55)	75 (53)	.052	NA	19 (24)	26 (47)	131 (61)	13 (50)	<.001***	BDI versus MDD
Yes	40 (66)	26 (62)	60 (45)	66 (47)			61 (76)	29 (53)	85 (39)	13 (50)		
Education level, *N* (%)												
<GCSE	3 (5)	1 (2)	2 (1)	1 (1)	.070	NA	2 (2)	1 (2)	2 (1)	1 (4)	.008**	BDI versus MDD
GCSE	7 (11)	3 (7)	5 (4)	13 (9)			18 (22)	5 (9)	19 (9)	6 (23)		
A‐level	19 (31)	15 (36)	30 (22)	39 (28)			26 (32)	25 (45)	56 (26)	7 (27)		
Undergraduate	17 (28)	18 (43)	58 (43)	64 (45)			20 (25)	18 (33)	92 (43)	9 (35)		
Postgraduate	15 (25)	5 (12)	39 (29)	24 (17)			14 (18)	6 (11)	47 (22)	3 (12)		
Employment status^b^, *N* (%)												
Employed/Self‐employed	45 (74)	20 (48)	83 (62)	82 (58)	.005**	BDI versus None	50 (62)	36 (65)	129 (60)	19 (73)	.913	NA
Maternity leave	0 (0)	0 (0)	0 (0)	0 (0)			1 (1)	0 (0)	1 (0)	0 (0)		
Student	9 (15)	15 (36)	44 (33)	54 (38)			15 (19)	14 (25)	60 (28)	3 (12)		
Retired	0 (0)	0 (0)	0 (0)	0 (0)			0 (0)	0 (0)	1 (0)	0 (0)		
Unemployed	7 (11)	7 (17)	7 (5)	5 (4)			14 (18)	5 (9)	25 (12)	4 (15)		
Self‐rated physical health, *N* (%)												
Poor	5 (8)	3 (7)	4 (3)	1 (1)	.028*	BDI versus MDD	4 (5)	3 (5)	6 (3)	0 (0)	.261	NA
Not great	18 (30)	7 (17)	14 (10)	22 (16)		BDI versus None	25 (31)	19 (35)	57 (26)	6 (23)		
Fair	22 (36)	11 (26)	53 (40)	49 (35)			32 (40)	19 (35)	83 (38)	10 (38)		
Good	12 (20)	17 (40)	51 (38)	53 (38)			18 (22)	14 (25)	60 (28)	7 (27)		
Very good	4 (7)	4 (10)	12 (9)	16 (11)			1 (1)	0 (0)	10 (5)	3 (12)		
Self‐rated mental health, *N* (%)												
Poor	10 (16)	6 (14)	6 (4)	4 (3)	<.001***	BDI versus None	24 (30)	10 (18)	38 (18)	2 (8)	.005**	BDI versus None
Not great	29 (48)	22 (52)	73 (54)	37 (26)		BDII versus None	45 (56)	32 (58)	124 (57)	10 (38)		
Fair	17 (28)	12 (29)	42 (31)	68 (48)		MDD versus None	10 (12)	10 (18)	51 (24)	13 (50)		
Good	5 (8)	2 (5)	13 (10)	31 (22)			1 (1)	3 (5)	3 (1)	1 (4)		
Very good	0 (0)	0 (0)	0 (0)	1 (1)			0 (0)	0 (0)	0 (0)	0 (0)		
Childhood trauma^c^, *N* (%)												
No	18 (30)	18 (43)	68 (51)	91 (65)	<.001***	BDI versus None	25 (31)	23 (42)	103 (48)	13 (50)	.026*	‐
Yes	42 (69)	24 (57)	60 (45)	45 (32)			55 (69)	31 (56)	111 (51)	11 (42)		
Prefer not to say	1 (2)	0 (0)	6 (4)	5 (4)			0 (0)	1 (2)	2 (1)	2 (8)		
Who diagnosed MDD, *N* (%)												
GP	NA	NA	NA	NA	NA	NA	56 (70)	40 (73)	192 (89)	24 (92)	.009**	BDI versus MDD
Psychiatrist	NA	NA	NA	NA			24 (30)	15 (27)	23 (11)	2 (8)		
Other medical professional	NA	NA	NA	NA			0 (0)	0 (0)	1 (0)	0 (0)		
Other/does not remember	NA	NA	NA	NA			0 (0)	0 (0)	0 (0)	0 (0)		
Age at first low mood episode, mean (*SD*), years	15.6 (4.7)	14.6 (4.3)	17.1 (5.0)	18.2 (5.8)	.001**	BDII < MDD	16.1 (6.1)	15.6 (6.3)	17.8 (6.5)	21.0 (6.9)	.002**	BDI < None
						BDII < None						BDII < None
Number of low mood episodes, *N* (%)												
0	1 (2)	3 (7)	3 (2)	22 (16)	<.001***	BDI versus None	0 (0)	0 (0)	0 (0)	0 (0)	.004**	‐
1	0 (0)	0 (0)	10 (7)	7 (5)		MDD versus None	1 (1)	2 (4)	8 (4)	4 (15)		
>1	60 (98)	39 (93)	121 (90)	112 (79)			79 (99)	53 (96)	208 (96)	22 (85)		
Number of elevated mood episodes, *N* (%)												
0	2 (3)	4 (10)	45 (34)	54 (38)	<.001***	BDI versus MDD	3 (4)	5 (9)	97 (45)	13 (50)	<.001***	BDI versus MDD
1	7 (11)	7 (17)	17 (13)	22 (16)		BDI versus None	3 (4)	0 (0)	20 (9)	2 (8)		BDI versus None
>1	52 (85)	31 (74)	72 (54)	65 (46)		BDII versus None	74 (92)	50 (91)	99 (46)	11 (42)		BDII versus MDD
												BDII versus None
PHQ−9 score^d^, mean (*SD*)	14.4 (5.0)	14.1 (4.2)	12.7 (4.7)	10.0 (4.1)	<.001***	BDI > None	16.4 (4.4)	16.2 (4.9)	13.8 (4.7)	12.4 (5.2)	<.001***	BDI > MDD
						BDII > None						BDI > None
						MDD > None						BDII > None
WEMWBS score^d^, mean (*SD*)	34.5 (7.6)	35.4 (7.3)	37.0 (7.3)	41.7 (7.5)	<.001***	BDI < None	32.4 (6.9)	33.2 (8.1)	35.1 (7.3)	38.4 (8.0)	.005**	BDI < None
						BDII < None						
						MDD < None						

Note

Shown are differences between the BD, MDD and low mood groups in self‐reported demographic and clinical characteristics of participants with no previous mood disorder diagnosis and in participants with a previous diagnosis of MDD. Please refer to Appendix S1 for the complete group comparison, including subthreshold BD and established BD groups. *p* values were obtained from the Kruskal–Wallis test for continuous variables and χ^2^ test for categorical variables, with Dunn's and pairwise χ^2^ post hoc tests, respectively. Bonferroni correction was applied to all possible group comparisons between the six CIDI diagnoses within each baseline group (i.e., 15 comparisons; Appendix S1).

Abbreviations: BD, bipolar disorder; CIDI, Composite International Diagnostic Interview; GCSC, General Certificate of Secondary Education; GP, general practitioner; MDD, major depressive disorder; NA, not applicable; PHQ‐9, Patient Health Questionnaire‐9; *SD*, standard deviation; WEMWBS, Warwick‐Edinburgh Mental Wellbeing Scale.

^a^ Past 12 months.

^b^ Current.

^c^ The presence of childhood trauma was evaluated using a single item about adverse childhood events up to the age of 10.

^d^ Past two weeks.

Of those who had been previously diagnosed with MDD, participants with newly diagnosed BDI were more likely to smoke relative to the MDD and subthreshold BD groups. Furthermore, individuals with BDI had lower levels of education in comparison to those with MDD, and rated their mental health as being worse relative to the low mood group. The MDD group was more likely to have been previously diagnosed with MDD by a GP, while BDI individuals were more likely to have been previously diagnosed with MDD by a psychiatrist. The majority (58%, 50/86) of individuals with BD who were diagnosed as MDD by a GP, for whom data about the onset of manic symptoms was available (data was missing for 10 individuals), had experienced manic symptoms before being diagnosed with MDD, while the majority (54%, 19/35; data missing for 4 participants) of individuals with BD diagnosed as MDD by a psychiatrist experienced manic symptoms after being diagnosed with MDD (Table 2). Participants with BD were younger when they experienced their first low mood episode relative to the low mood group. In addition, individuals with BD and MDD with subthreshold BD reported having experienced more episodes characterized by elevated mood in comparison to MDD and low mood groups. Similarly, those with subthreshold BD reported having experienced more elevated mood episodes relative to the MDD group. Individuals with BDI reported higher depression severity in comparison to the MDD and low mood groups. Similarly, individuals with BDII scored higher in depression severity relative to the low mood group. Furthermore, individuals with BDI reported poorer wellbeing in comparison to those with low mood. There were no other significant group differences in participants with a previous MDD diagnosis.

**TABLE 2:** Relative timing of MDD diagnosis and first (hypo)manic symptoms in BD patients previously diagnosed with MDD (*N* = 135)

Medical professional	(Hypo)manic symptoms before MDD diagnosis	(Hypo)manic symptoms after MDD diagnosis	NA
GP	50	36	10
Psychiatrist	16	19	4
Other medical professional	0	0	0
Other/does not remember	0	0	0

Note

‘Medical professional’ refers to a professional who made the MDD diagnosis.

Abbreviations: BD, bipolar disorder; GP, General Practitioner; MDD, major depressive disorder; NA, not available.

Finally, with regards to the BD validation group, the CIDI showed 89% sensitivity in identifying participants with a previous diagnosis of BD. The majority of participants with an existing diagnosis of BD reported a previous diagnosis of MDD (81%, 44/54) and had been treated with antidepressant medication (89%, 48/54). As expected, MDD diagnoses in this group were made mostly by a GP (52%, 23/44), while the BD diagnoses were made primarily by a psychiatrist (87%, 47/54). Approximately half (47%, 18/38) of participants with established BD diagnosed initially as MDD, for whom data about the onset of manic symptoms was available (data missing for 6 participants), had experienced first manic symptoms before being diagnosed with MDD (Table 3). The average time interval (±standard deviation) between the diagnosis of MDD and the diagnosis of BD was 5.4 ± 5.9 years, and the average duration of BD diagnosis was 7.3 ± 6.6 years.

**TABLE 3:** Relative timing of MDD diagnosis and first (hypo)manic symptoms in established BD patients initially diagnosed with MDD (*N* = 44)

Medical professional	(Hypo)manic symptoms before MDD diagnosis	(Hypo)manic symptoms after MDD diagnosis	NA
GP	11	8	4
Psychiatrist	6	9	1
Other medical professional	0	2	1
Other/does not remember	1	1	0

Note

‘Medical professional’ refers to a professional who made the MDD diagnosis.

Abbreviations: BP, bipolar disorder; GP, General Practitioner; MDD, major depressive disorder; NA, not available.

## 4 DISCUSSION

The aim of the present study was to explore the prevalence and characteristics of mood disorders in the Delta Study population. Overall, the present findings support the notion that all individuals presenting with depressive symptoms should be carefully assessed and monitored. Digital technologies, such as those utilized in the Delta Study, could aid in this process by offering an innovative, time‐efficient and cost‐effective means to improve mental healthcare provision, facilitating, in turn, biological disease understanding and patient stratification, and allowing for personalized treatment plans and strategies. Of the symptomatic individuals with no previous diagnosis of mood disorder, the prevalence rates of MDD and BD were 36% and 24%, respectively, while 33% did not meet criteria for either disorder. Individuals who were newly diagnosed with BDI were more likely to have suffered childhood trauma relative to the low mood group. Although the online mental health questionnaire employed in the Delta Study did not capture different forms of childhood trauma (e.g., emotional, physical, or sexual abuse), research has demonstrated that emotional abuse may be particularly associated with BD (Janiri et al., 2015; Etain et al., 2010). Regarding depressive states, those with BDII were significantly younger when they experienced their first low mood episode relative to the MDD and low mood groups, consistent with an earlier age at onset of BD compared to MDD (Tondo et al., 2010). Interestingly, individuals diagnosed with BDI and MDD did not differ in the number of low mood episodes and there were no differences in self‐rated quality of mental health and wellbeing, as well as in depression severity, between the BD and MDD groups. As expected, participants with BDI reported having experienced a higher number of episodes characterized by elevated mood relative to both the MDD and low mood groups, in line with the diagnostic criteria for the condition (American Psychiatric Association, 2013; World Health Organization, 2019). Similarly, the number of elevated mood episodes was higher in BDII and subthreshold BD in comparison to those with low mood. These findings emphasize the importance of assessing for (hypo)manic symptoms in patients presenting with low mood, particularly in light of the largely indistinguishable differences in depressive episodes between BD and MDD.

Regarding participants who had been previously diagnosed with MDD, the findings from the current study revealed that 31% met the criteria for BD according to the CIDI. The prevalence of BD previously diagnosed as MDD in the current study was consistent with figures found in the literature, which vary between 10% and 50% (Cassano et al., 1992; Ghaemi et al., 2002; Benazzi, 1997; Hirschfeld et al., 2003; Shen et al., 2018). This variation could be attributed to differences in recruitment and sampling procedures. Notably, an exploration of symptom‐ and patient‐level data revealed that BDI individuals with a previous diagnosis of MDD were less educated relative to those whose MDD diagnosis was confirmed by the CIDI. Furthermore, the finding that individuals with BDI were more likely to smoke in comparison to the MDD group is in line with Li et al. (Li et al., 2017). While we found no significant differences in depression severity between BD and MDD in those with no previous mood disorder diagnosis, participants with BDI previously diagnosed as MDD scored higher on the PHQ‐9 relative to MDD and low mood individuals, potentially reflecting the effects of receiving ineffective treatment (e.g., antidepressant monotherapy) and the lack of support due to incorrect diagnosis. Similarly, individuals with BDII previously diagnosed as MDD exhibited elevated depression severity relative to the low mood group. These findings are largely in line with studies revealing a direct association between depression severity and BD (Strober & Carlson, 1982; Holma et al., 2008). In accordance with previous research (Hirschfeld et al., 2005) and, in part, with the results from the baseline low mood group, individuals with BD reported being younger when they experienced their first low mood episode relative to the low mood group, and individuals with BDI reported poorer quality of mental health and wellbeing. Furthermore, in line with the findings in individuals with no previous mood disorder diagnosis, individuals with BD reported having experienced more episodes characterized by elevated mood relative to the MDD and low mood groups. Importantly, 70% of BDI individuals who had been diagnosed with MDD were assessed by a GP, with the majority of these reporting having experienced episodes of elevated mood *prior* to being assessed. These findings stress the importance of asking about (hypo)manic symptoms in the primary care setting, with the presence of elevated mood warranting further evaluation by a psychiatrist, as recommended by the National Institute for Health and Care Excellence (NICE) (National Collaborating Centre for Mental Health (UK), 2014).

Importantly, the current research has a number of limitations. Firstly, due to the cross‐sectional nature of the study design, we were not able to examine longitudinal changes in symptoms and diagnoses. Secondly, no means were available to validate the information self‐reported by participants, and some important information was not collected or only partially collected. Furthermore, the CIDI was 89% sensitive in detecting previously diagnosed participants with BD, hence introducing diagnostic uncertainty. Finally, the recruitment material targeted individuals who were concerned about their mood disorder diagnosis, particularly those with a high risk of BD. As such, there is likely to be a recruitment bias and the findings from the current study may not generalize to the broader population or to those with more severe forms of psychopathology. Despite these limitations, this is the first diagnostic study, to our knowledge, to extensively evaluate a large online cohort of individuals suffering from mood disorders or low mood. The current findings have important clinical implications and indicate an urgent need for innovative, accessible, time‐ and cost‐effective alternatives to conventional, interview‐based diagnostic methods. Further research is necessary in order to explore the potential of using an online mental health questionnaire as a means for aiding in clinical decision‐making and improving management of mood disorders in healthcare settings.

## 5 CONCLUSION

Taken together, the key findings from the current research are 2‐fold. First, given that depressive episodes in BD and MDD patients with no previous mood disorder diagnosis were largely indistinguishable, standard screening practices must go beyond brief symptom‐count checklists, such as the PHQ‐9 (Kroenke et al., 2001), when assessing the symptoms of those presenting with depressive symptoms. Indeed, a careful and comprehensive evaluation of mood states in these individuals is warranted, with a recent machine learning study demonstrating that self‐reported symptoms of elevated mood and grandiosity, as well as increased talkativeness and recklessness, can offer excellent discriminatory performance when distinguishing between BD and MDD (Tomasik et al., 2021). While time is a premium in primary care settings, with 50% of the global population spending five minutes or less per visit with their primary care physician (Irving et al., 2017), a highly scalable, low‐cost online mental health questionnaire has the potential to facilitate the identification of BD and MDD in those presenting with low mood. Second, in light of the potential for the misdiagnosis of BD, all individuals diagnosed with MDD should be closely monitored and managed, with antidepressant‐induced (hypo)mania and non‐response to antidepressant medication warranting specialized evaluation by a psychiatrist to rule out a BD diagnosis.

## CONFLICT OF INTEREST

SB is a director of Psynova Neurotech Ltd. and Psyomics Ltd. SB, NMK, DC, GBO, LPF and EB have financial interests in Psyomics Ltd. SB, PE and TO have received payments from the University of Cambridge for licensing of data from the Delta Study. All other authors declare no competing interests.

## AUTHOR CONTRIBUTIONS

SB and DC conceived the study and conceptualized and supervised the development of the online mental health assessment. SB, DC, GBO, TO, JDC, SYHS, LPF, LVF and EB were involved in the design of the study. GBO, TO and PE collected the online mental health assessment data. GBO developed the diagnostic algorithm. JT processed and analyzed the data and produced the figures, with input from NMK and SB. NMK and JT drafted the manuscript, with contributions from SB. All authors were involved in reviewing the final version of the manuscript. PE provided IT support. LPF provided regulatory advice.

### PEER REVIEW

The peer review history for this article is available at https://publons.com/publon/10.1002/brb3.2167.

## Supporting information

Appendix S1

## Data Availability

Data and scripts used in this study are available upon approval from the corresponding authors at sb209@cam.ac.uk or jt455@cam.ac.uk. Data availability is subject to data license agreements between the University of Cambridge and Psyomics Ltd.
